# Perceived social support in pregnant adolescents in Mersin area in Turkey

**DOI:** 10.12669/pjms.341.14221

**Published:** 2018

**Authors:** Mine Yurdakul

**Affiliations:** Mine Yurdakul, PhD. Assistant Professor, Mersin University School of Health, Mersin, Turkey

**Keywords:** Adolescent pregnancy, Perceived social support, Social support, Teenage pregnancy

## Abstract

**Objective:**

The study examines the level and source of perceived social support in pregnant adolescents and the factors related to their perception of social support.

**Methods:**

This descriptive study was conducted with the voluntary participation of 127 adolescent pregnant females who visited the Gynecology and Pediatric Hospital in Mersin, Turkey. The data were collected based on the participants' self-expression, using the Socio-demographic Information Form and Multidimensional Scale of the Perceived Social Support.

**Results:**

The average age of the pregnant adolescents was 18 years. Approximately one-fifth of all participant females were either illiterate or had dropped out of the primary school. All pregnant adolescents were housewives with a low economic status. Findings pertaining to the participants'fertility showed that 69.3% were primiparous, 24.4% had at least one living child. The mean score for pregnant adolescents' perception of social support was 50.79±8.72. The mean score on the subscales was 23.32±3.23 for family support; 16.17±4.35 for friend support; and 12.29 ± 5.54 for special person support.

**Conclusion:**

Pregnant adolescents had a low perception of social support. Families were found to be the most common source of social support available to pregnant adolescents, and they lacked the support from their friends and other special people.

## INTRODUCTION

Adolescence is the transformative from childhood to adulthood. In this period, individuals, particularly girls, rapidly grow and develop through biological, psychological and social changes. This is a special period that influences the adolescent's current and future health, depending on their chosen set of behavior and life styles.^1^,^2^ The World Health Organization (2014) identifies adolescence as the period between 10-19 years.^3^ Adolescent pregnancy is generally unintended and occurs due to early marriage or early sexual intercourse. Approximately 16 million young women inthe 15-19 age group give birth every year, which constitute 11% of all births around the world.^3^ In Turkey, approximately 5% of the adolescent girls are known to give birth.^4^ Adolescent pregnancies lead to negative obstetric and neonatal consequences due to biological, psychological, and social factors. They increase the risk for maternal and infant mortality.^5^ Moreover, adolescent pregnancies have social consequences including limitations to girls' education, domestic violence and suicide.^6^ Thus, it is crucial to provide social support in addition to health services for pregnant adolescents.

Social support systems are defined as all forms of voluntary interpersonal relationships geared towards providing financial, emotional, and cognitive aids.^6^,^7^ An individual's family, friends, neighbors, and teachers constitute the sources of social support. Social support can be classified into three groups: financial, emotional, and cognitive. Financial support involves providing money, food and help in domestic work. Emotional support includes accepting and meeting individuals' basic social needs such as love, attention, trust, and the sense of belonging to a group. Cognitive support refers to providing information and support to help individuals in solving their problems.^8^

Many studies highlight the positive effects of social support on health.^8^-^10^ They indicate that receiving sufficient social support has positive results which contribute to the improvements in adolescent's health, have a positive effect on the adaptation process of the adolescent to motherhood.^6^ Adolescents ‘cognitive abilities, communication and social skills differ from those of adults. This difference affects not only their motherhood roles but also their skills for providing social support.

Early pregnancies in Turkey can frequently be attributed to early marriages.^4^ It is known that pregnant adolescents usually do not plan their pregnancies and have limited access to the social and economic resources.^6^ Therefore, pregnant adolescents may need more social support than adult pregnant. Midwives and nurses should consider this fact and evaluate the social support of pregnant adolescents. There are many studies about the obstetric and neonatal consequences of adolescent pregnancies. However, there are a limited number of studies focusing on the antepartum social support for pregnant adolescents. Specifically, it may be of utmost importance to acquire information about pregnant adolescents' perception of social support and its sources.

This study aimed to analyze pregnant adolescents' perception of social support and the related factors. The second aim of the study was to find out the pregnant adolescents ‘sources of social support.

## METHODS

The study was carried out in the clinics at the Gynecology and Pediatric Hospital in Mersin, Turkey. This hospital, in Mersin city center, provides health services to individuals from different socioeconomic groups with its large capacity. The study sample consisted of 118 pregnant adolescents. This number was obtained through the minimum required sample size calculation with ±3 standard deviation and 95% confidence for seven-point Likert type scales.^11^ However, the study included 127 pregnant adolescents. The data were collected by the interviewer, trained in the study subject and the data collection process, between December 1, 2015 and June 31, 2016, and controlled and assessed by the researcher.

The Socio-demographic information form was prepared by the researcherincluding 18 questions about the socio-demographic data, to determine the pregnant adolescents' perception of social support The Multidimensional Scale of Perceived Social Support (MSPSS), was administered to determine the pregnant adolescents' perception of social support. The MPSS was developed by Zimet et al. in 1988, and adapted to Turkish by Eker et al. It was restructured in 2001 to distinguish the terms “family” and “special person”.^12^,^13^ It includes the following subscales of perceived social support: family, friend, and a special person (such as neighbors or teachers). It has 12 items; four items in each subscale, listed as follows: “Family” (items 3, 4, 8 and 11), “Friend” (items 6, 7, 9 and 12) and “Special person” (items 1, 2, 5 and 10). Each item is ranked using the seven-point Likert type scale. The subscale scores are obtained by summing the scores of four items in each subscale. The sum of all subscale scores yields the total scale score. The minimum and maximum scores that can be obtained from the subscales are 4 and 28, respectively. The minimum and maximum total score of the scale is 12 and 84, respectively. High scores indicate a high level of perceived social support; whereas low scores indicate a lack of social support. Eker et al. (2001) found the total Cronbach's alpha coefficient of the MSPSS to be 0.89, and the Cronbach's alpha coefficients for the subscales to are 0.85 for the family, 0.88 for the friend, and 0.92 for the special person subscales. In this study, the total Cronbach's alpha coefficient of the MSPSS was 80.3, while the subscales' Cronbach's alpha coefficients were 80.9 for the family, 87.7 for the friend, and 92.1 for the special person subscales. MSPSS scale was used since it can be easily administered and understood by laypeople.

In data analysis, the descriptive statistics were presented through frequency and percentage for the categorical data, and through minimum, maximum, average, and standard deviation for the continuous variable. The student's t-test was used to compare the means of two independent groups for continuous variables, and the One-way Analysis of Variance (ANOVA) was used to compare the means of more than two independent groups. The LSD test was utilized as the Post Hoc test. Statistical significant level was accepted to be 0.05.

### Ethical Considerations

A written permission l was obtained from the institution where the study was conducted; the ethical approval was obtained from the Clinical Research Ethics Committee of Mersin University. Oral consent was obtained from the participants who were informed about the objective of this study.

## RESULTS

The average age of the pregnant adolescents was 18.09 ± 0.99 (min15/max19). Approximately one-fifth of them was illiterate or had dropped out of the primary school. A majority of them (91.3%) had social security and civil marriages. None of the pregnant adolescents lived alone; and 59% of them lived in nuclear families. All pregnant adolescents were housewives and had a low economic status (income is less than expenses) (data not shown). Of them, 69.3% were pregnant for the first time, 25.5% for the second time, and 5.5% for the third time. The average week of pregnancy was found to be 33.01±7.21 (12–41). Of the adolescents,24.4% had at least one living child,70.1% reported their pregnancy as intended and 88.2% of the pregnant adolescents received prenatal care at least once (Table-I).

**Table-I:** Pregnant adolescents' descriptive characteristics

Characteristics	Frequency	Percent (%)
**Average Age** 18.09 ± 0.99 (15-19)		
*Education*	
Illiterate	14	11.0
Primary School (Incomplete)	10	7.9
Primary School	87	68.5
High School	16	12.6
*Social Security*	
Yes	116	91.3
No	11	8.7
*Civil Marriage*	
Yes	94	74.0
No (Religious marriage)	33	26.0
**Average age at marriage** 17.83±12.86 (14-19)		
*Family Type*	
Nuclear family	75	59.1
Extended family	52	40.9
*Total Number of Pregnancies*		
*First pregnancy*		
2	88	69.3
3	32	25.2
88	7	5.5
*Living Child*	
None	96	75.6
One and more	31	24.4
*Current pregnancy*	
Intended	89	70.1
Unintended	38	29.9
*Prenatal Care*	
Received	112	88.2
Did not receive	15	11.8

Total	127	100.0

**Fig. 1:**
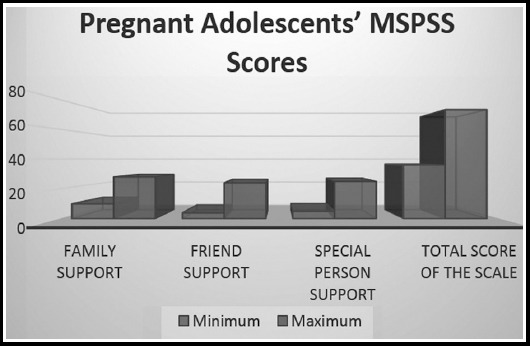
The pregnant adolescents' the minimum-maximum scores on the scale.

**Fig. 2:**
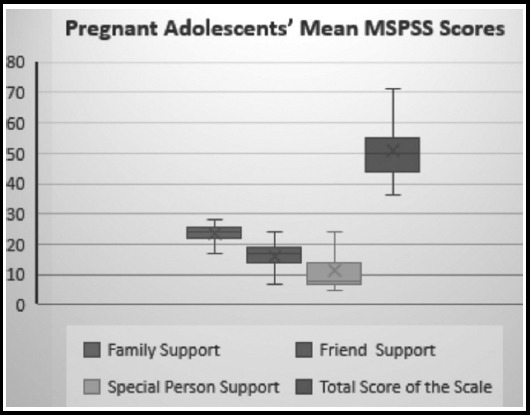
Pregnant adolescents' mean MSPSS scores

The pregnant adolescents' mean MSPSS score and the minimum-maximum scores on the scale are presented in Fig.1. The pregnant adolescents' mean MSPSS score was 50.79±8.72. Their mean scores on the subscales were 23.32 ± 3.23 for family support, 16.17 ± 4.35for friend support, and 12.29 ± 5.54forspecial person support (Fig.2).

Comparison of the MSPSS scores according to the pregnant adolescents' descriptive characteristics is shown in Table-II. A statistically significant difference was not found among the mean scores on the family support subscale (p=0.383), the mean scores on the friend support subscale (p=0.478) and mean scores on the total score of the scale (p=0.175) based on the education level. However a statistically significant difference was found between the mean scores on the special person support subscale based on education groups (p=0.013). To determine which group had difference, advanced analysis had been applied. The difference was between those having the illiterate group with primary school (p=0.021), and illiterate group with high school (p=0.019). The perception of the special person support of the pregnant adolescents who graduated from high school was high.

**Table-II:** Comparison of the MSPSS scores according to the pregnant adolescents' descriptive characteristics.

Characteristics	N	Family Support Mean ±SD	Friend Support Mean.± SD	Special Person Support Mean ± SD	Total Scale Score Mean ±SD
*Education*					
Illiterate	14	22.00±4.72	16.56±4.16	9.75±4.07	54.43±12.03
Primary School(Incomplete)	10	23.50±2.80	14.50±4.17	9.80±3.99	47.80±7.15
Primary School	87	23.51±3.07	16.44±4.03	11.06±5.59	51.00±8.56
High School	16	23.57±2.21	15.29±6.38	15.57±5.98	48.31±6.19
		p=0.383	p=0.478	p=0.013	p=0.175
*Social Security*					
Yes	116	23.37±3.06	15.93±4.17	10.68±5.04	49.98±8.04
No	11	22.82±4.79	18.73±5.59	17.73±6.66	59.27±11.32
		p=0.589	p=0.041	p<0.001	p=0.001
*Family type*					
Nuclear family	75	23.75±2.94	15.75±4.39	10.41±4.95	49.91±8.20
Extended Family	52	22.71±3.54	16.79±4.26	12.56±6.12	52.06±9.36
		p=0.075	p=0.186	p=0.031	p=0.173
*Number of Pregnancies*					
1	88	23.60±3.11	16.43±4.57	11.88±5.82	51.91±9.09
2	32	22.78±3.70	15.44±3.95	9.66±4.48	47.88±7.20
3	7	22.29±1.98	16.29±3.20	11.43±5.59	50.00±8.33
		p=0.332	p=0.544	p=0.152	p=0.078
		p=0.295	p=0.496	p=0.543	p=0.378

A statistically significant difference was not found between the mean scores on the family support subscale based on social security groups. On the other hand, a statistically significant difference was found among the mean scores on the friend support subscale (p=0.041), the mean scores on the special person support subscale (p<0.001), and the mean scores on the total scores of the scale (p=0.001) based on social security groups.

A statistically significant difference was not found between the mean score based on the family (p=0.075) and friend support subscale (p=0.0186) based on the family types. However, statistically significant difference was found between the mean scores on the special person support subscale based on the family types (p=0.031). Accordingly adolescents who live in the extended families have a high perception of social support from special person like neighbors or teachers. Although there was no statistically significant difference between the mean total scores based on the family types, the pregnant adolescents living in extended families obtained a higher mean score on the social support subscale. The mean scores on the social supports subscale were not significantly related to the number of pregnancies, pregnancy week or receiving prenatal care (p>0.05).

## DISCUSSION

Adolescent pregnancies constitute a contemporary problem for both countries and the international institutions since they are more frequently observed and have negative effects on the health of the mother and child. Studies on pregnant adolescents indicate that adolescent pregnancy is closely related to low economic conditions.^14^ The pregnant adolescents in the present study had similar socioeconomic characteristics. These studies report that most of such pregnancies are unintended and unplanned, forcing young women to drop out of school.^10^,^15^ In contrast to the previous studies, the present study indicates that most of the pregnant adolescents (70.1%) wished their current pregnancy. This may be because early marriages and having children at early ages are widely accepted by the society in the region.

The mean MSPSS score of pregnant adolescents was 50.79±8.72. The mean scores of the social support pregnant adolescents received from their families, friends, and a special person were 23.32±3.23, 16.17±4.35 and 12.29±5.54, respectively. The pregnant adolescents perceived the most social support coming from their families, and less social support coming from their friends and special persons. Mermer et al. (2010) investigated the social support received during and at the end of pregnancy and found the mean MSPSS score to be 66.70±15.54.^7^ Metin and Pasinlioglu et al. (2016) examined the relationship between social support for pregnant women and prenatal bonding, and found the mean total score to be 63.88±14.49.^16^ In comparison with their study, which used the same scale, pregnant adolescents' perception level of social support was low in the present study. Moreover, the findings of some other studies support our findings. For example, Babington et al. (2015) found that pregnant adolescents' perceptions of social support are lower than the adolescents who are not pregnant.^15^ The same study also mentioned the responsibilities of mothers, grandmothers, and sisters in providing baby care. McVeigh and Smith (2010) conducted study on adolescent and adult mothers and showed that although satisfaction from the social support was significantly low for both groups, partner support was higher for young mothers.^17^ Longston et al. (2005) determined that pregnant adolescents receivemore support from their mothers than from their fathers. They also stated that financial support was the main difficulty for pregnant adolescents and the primary issue that should be supported.^6^ Letourneau et al. (2004) reported that the most important source of social support for adolescent mothers are their mothers and family members, followed by the father of their baby.^9^ In line with the previous studies, the present study found that while the adolescents had a perception of low level of social support, they received the highest level of social support from their families. Perceived social support from friends and special person's was also considerably low in this study, which could be attributed to the fact that almost half of the adolescents lived in extended families and had limited connections with their social environment.

It is reported that people with higher education levels tend to make more use of their support sources, as they might have developed skills to mobilize social support sources.^7^ Likewise, Metin and Pasinlioglu (2016) argued that pregnant women's perception of social support becomes higher as their education level rose to the university level. In this study, there were no significant difference between education status, family, friend and total mean scale score on MSPSS. However adolescents who have higher education status have high perception of special person support. This result augments the findings of the previous study.^7^,^16^

Having a social security or health insurance helps a person to feel safe and effect positively about the perception of social support. However, in this study, adolescent who do not have social security performed higher perception of special people, friend and mean total score of the scale was found high in this group. This result may be explained by adolescents' receiving more support or being directed to the social welfare institutions by their friends, teachers or neighbors. Because, traditionally, people in need are supported; thus, adolescent pregnancy has been widely accepted.

Pregnant adolescents' family structure affects their perception of social support. Studies on this subject report that the mean social support scores are higher for nuclear families than for extended families.^7^ In contrast to previous studies, the social support scores of the pregnant adolescents living in extended families were higher in the present study. Extended family type refers to the families with many family members; and the increased number of family members may increase the perception of support. In addition, adolescents who marry at early ages usually live with the family of their spouses, and make use of this family's financial and other opportunities, which may also increase the perception of social support.

First pregnancies are generally a pleasing and exciting situation for both the expectant parents and their families. Professional support from midwives and social support from the relatives and friends are extremely important for women experiencing their first pregnancy. Although these support types differ, they are important for women to have a positive birth experience.^9^ More support are provided by the spouse, family and health personnel during the first pregnancy.^11^ This study showed no statistically significant difference between the number of pregnancies and the mean social support score; however, the primiparous adolescents' total score was relatively higher.

## CONCLUSION

Pregnant adolescents in this study needed social support from their friends and special persons. During prenatal care services, pregnant adolescents should be considered together with their social surroundings and their social support sources and social support status should be determined accordingly. Pregnant adolescents and their families should be informed about the importance of social support and the ways to mobilize social support sources.

## References

[1] Solt A, Yazici S (2015). Adolescent pregnancies. J Health Sci Prof.

[2] Cinar N, Hira S (2017). Adolescent mother. J Human Rhythm.

[3] World Health Organization (2015). Maternal, newborn, child and adolescent health. Adolescent pregnancy.

[4] TPHR (Turkey Population and Health Research) (2013). Hacettepe University Institute of Population Studies.

[5] Mouli VC, Camacho AV, Michaud PA (2013). WHO Guidelines on preventing early pregnancy and poor reproductive outcomes among adolescents in developing countries. J Adolesc Health.

[6] Logsdon CM, Gagne P, Hughes T, Patterson J, Rakestraw V (2005). Social support during adolescent pregnancy: piecing together a quilt. J Obstet Gynecol Neonatal Nurs.

[7] Mermer G, Bilge A, Yucel U, Ceber E (2010). Evaluation of perceived social support levels in pregnancy and postpartum periods. J Psychiatr Nurs.

[8] Backstrom C, Larsson T, Wahlgren E, Golsater M, Martensson LB, Thorstensson S (2017). “It makes you feel like you are not alone”: Expectant first – time mothers'experiences of social support within the social network, when preparing for childbirth and parenting. Sex Reprod Healthc.

[9] Letourneau NL, Stewart MJ, Barnfather AK (2004). Adolescent mothers: support needs, resources, and support-education interventions. J Adolesc Health.

[10] Peter PJ, Mola CL, Matos MB, Coelho FM, Pinheiro KA, Silva RA (2017). Association between perceived social support and anxiety in pregnant adolescents. Rev Bras Psiquiatr.

[11] Bartlett JE, Kotrlik JW, Higgins CC (2001). Organizational research: Determining appropriate sample size in survey research. Information Technology. Learn Perform J.

[12] Zimet GD, Dahlem NW, Zimet SG, Farley GK (1988). The Multidimensional scale of perceived social support. J Pers Assess.

[13] Eker D, Arkar H, Yaldız H (2001). Factorial structure, validity, and reliability of revised form of the multidimensional scale of perceived social support. Turk J Psychiatry.

[14] Sedgh G, Finer LB, Bankole A, Eilers MA, Singh S (2015). Adolescent pregnancy, birth, and abortion rates across countries: Levels and recent trends. J Adolesc Health.

[15] Babington LM, Malone L, Kelley BR (2015). Perceived social support, self-esteem, and pregnancy status among Dominican adolescents. App Nurs Res.

[16] Metin A, Pasinlioglu T (2016). The Relationship between perceived social support and prenatal attachment in pregnant women. Int Refereed J Gynaecol Maternal Child Health.

[17] McVeigh C, Smith M (2000). A Comparison of adult and teenage mother's self-esteem and satisfaction with social support. Midwifery.

